# Empirical analyses and simulations showed that different machine and statistical learning methods had differing performance for predicting blood pressure

**DOI:** 10.1038/s41598-022-13015-5

**Published:** 2022-06-03

**Authors:** Peter C. Austin, Frank E. Harrell, Douglas S. Lee, Ewout W. Steyerberg

**Affiliations:** 1 ICES, G106, 2075 Bayview Avenue, Toronto, ON M4N 3M5 Canada; 2 Department of Health Policy, Management and Evaluation, University of Toronto, Toronto, ON Canada; 3 Schulich Heart Research Program, Sunnybrook Research Institute, Toronto, ON Canada; 4 Department of Biostatistics, Vanderbilt University School of Medicine, Nashville, TN USA; 5 Faculty of Medicine, University of Toronto, Toronto, ON Canada; 6 Department of Biomedical Data Sciences, Leiden University Medical Centre, Leiden, The Netherlands

**Keywords:** Epidemiology, Outcomes research, Translational research

## Abstract

Machine learning is increasingly being used to predict clinical outcomes. Most comparisons of different methods have been based on empirical analyses in specific datasets. We used Monte Carlo simulations to determine when machine learning methods perform better than statistical learning methods in a specific setting. We evaluated six learning methods: stochastic gradient boosting machines using trees as the base learners, random forests, artificial neural networks, the lasso, ridge regression, and linear regression estimated using ordinary least squares (OLS). Our simulations were informed by empirical analyses in patients with acute myocardial infarction (AMI) and congestive heart failure (CHF) and used six data-generating processes, each based on one of the six learning methods, to simulate continuous outcomes in the derivation and validation samples. The outcome was systolic blood pressure at hospital discharge, a continuous outcome. We applied the six learning methods in each of the simulated derivation samples and evaluated performance in the simulated validation samples. The primary observation was that neural networks tended to result in estimates with worse predictive accuracy than the other five methods in both disease samples and across all six data-generating processes. Boosted trees and OLS regression tended to perform well across a range of scenarios.

## Introduction

Clinical investigators are increasingly interested in using machine learning (ML) methods to predict patient outcomes. There are ongoing efforts to assess which method is best for use in clinical medicine. In reviewing 71 studies that used both conventional statistical models and ML methods for estimating the probability of the occurrence of a binary outcome, Christodoulou et al.^1^ found that, in those comparisons that were at low risk of bias, there was, on average, no difference in the discriminative ability of the different methods. However, in those comparisons that were at high risk of bias, ML methods tended to have better discrimination. In a study in which they fit both random forests and logistic regression models in 243 datasets, Couronné et al.^2^ found that, on average, random forests had better discrimination than logistic regression (mean increase in c-statistic: 0.041). In a systematic review that included 10 studies, Hassanipour et al.^3^ found that artificial neural networks had better discrimination than logistic regression for predicting outcomes in trauma patients (pooled estimate of the c-statistic: 0.91 for neural networks vs. 0.89 for logistic regression). Finally, in a review of 20 clinical studies that compared the performance of standard statistical models with that of ML methods for predicting either mortality or readmission in patients hospitalized with heart failure, Shin et al.^4^ found that ML methods tended to have better discrimination than did standard statistical methods. Three of the above four studies focused on binary outcomes, while that of Shin and colleagues considered both binary and time-to-event outcomes. The relative performance of ML methods and conventional statistical methods for predicting continuous outcomes has received substantially less attention. In the current study we focus on prediction of a specific continuous outcome important in clinical medicine: systolic blood pressure.

Our objective was to compare the relative performance of ML methods with that of conventional statistical learning methods for predicting discharge blood pressure in patients hospitalized with cardiovascular disease. We considered linear regression estimated using ordinary least squares (OLS), the lasso, ridge regression, boosted regression trees, random forests, and artificial neural networks. The paper is structured as follows: In “Empirical analyses on the relative performance of methods for predicting blood pressure”, we conduct a series of empirical analyses using data on patients hospitalized with either acute myocardial infarction (AMI) or congestive heart failure (CHF). We compare the performance of the six different learning methods for predicting patients’ systolic blood pressure at hospital discharge in validation samples. In “Monte Carlo simulations for comparing the relative performance of different prediction methods”, we describe a series of Monte Carlo simulations motivated by the empirical analyses conducted in the previous section. We examined the effect of different data-generating processes on the relative performance of the six different prediction methods. Each data-generating process was based on a different fitted learning method. Finally, in “Discussion”, we summarize our findings and place them in the context of the existing literature.

## Empirical analyses on the relative performance of methods for predicting blood pressure

We conducted a set of empirical analyses to compare the performance of different machine and statistical learning methods in two different disease groups: patients hospitalized with acute myocardial infarction (AMI) and patients hospitalized with congestive heart failure (CHF). In each disease group we examined the ability of different methods to predict a patient’s systolic blood pressure at hospital discharge. Model performance was assessed using independent validation samples.

### Data sources

We used data from a study that collected data on patients hospitalized with either acute myocardial infarction (AMI) or congestive heart failure (CHF) during two different time periods^5^. We considered each disease (AMI vs. CHF) separately. For the AMI patients, the derivation sample consisted of 8145 patients discharged alive from hospital between April 1, 1999 and March 31, 2001, while the validation sample consisted of 4444 patients discharged alive from hospital between April 1, 2004 and March 31, 2005. For the CHF patients, the derivation sample consisted of 7156 patients discharged alive from hospital between April 1, 1999 and March 31, 2001, while the validation sample consisted of 6818 patients discharged alive from hospital between April 1, 2004 and March 31, 2005. Thus, the derivation and validation samples came from distinct time periods. Data on patient demographics, vital signs and physical examination at presentation, medical history, and results of laboratory tests were collected for these samples. For the current study, the outcome was a continuous variable denoting the patient’s systolic blood pressure at the time of hospital discharge.

We considered 33 candidate predictor variables in the AMI sample and 28 candidate predictor variables in the CHF sample (Table 1 (AMI sample) and Table 2 (CHF sample) for a listing of these variables). These variables consisted of demographic characteristics, presentation characteristics, vital signs on hospital presentation, classic cardiac risk factors, comorbid conditions, laboratory tests, electrocardiogram results, and signs and symptoms^6–8^. Baseline characteristics in the two derivation samples and the two validation samples are reported in Table 1 (AMI sample) and Table 2 (CHF sample). Differences in covariates between derivation and validation samples were tested using a t-test for continuous covariates and a Chi-squared test for binary variables.

**Table 1:** Baseline characteristics of patients in the AMI derivation and validation samples.

Variable	Derivation sample (N = 8145)	Validation sample (N = 4444)	P-value
**Outcome variable**			
Discharge systolic blood pressure	120.40 ± 19.69	122.48 ± 20.60	< 0.001
**Demographic characteristics**			
Age	66.51 ± 13.58	69.13 ± 14.32	< 0.001
Female	2792 (34.3%)	1709 (38.5%)	< 0.001
**Vital signs on hospital presentation**			
Systolic blood pressure	148.87 ± 31.15	144.64 ± 31.24	< 0.001
Diastolic blood pressure	83.86 ± 18.46	80.39 ± 18.42	< 0.001
Heart rate	83.61 ± 23.77	85.72 ± 23.74	< 0.001
Respiratory rate	20.86 ± 5.45	20.41 ± 5.32	< 0.001
**Presentation characteristics**			
Cardiogenic shock	56 (0.7%)	< = 5	***
Acute congestive heart failure/pulmonary edema	389 (4.8%)	293 (6.6%)	< 0.001
**Classic cardiac risk factors**			
Diabetes	2072 (25.4%)	1268 (28.5%)	< 0.001
Hypertension	3731 (45.8%)	2658 (59.8%)	< 0.001
Current smoker	2753 (33.8%)	1208 (27.2%)	< 0.001
Dyslipidemia	2597 (31.9%)	2004 (45.1%)	< 0.001
Family history of coronary artery disease	2603 (32.0%)	1377 (31.0%)	0.262
**Comorbid conditions**			
Cerebrovascular accident/transient ischemic attack	772 (9.5%)	583 (13.1%)	< 0.001
Angina	2685 (33.0%)	1361 (30.6%)	0.007
Cancer	225 (2.8%)	80 (1.8%)	< 0.001
Dementia	250 (3.1%)	267 (6.0%)	< 0.001
Peptic ulcer disease	452 (5.5%)	226 (5.1%)	0.27
Previous AMI	1824 (22.4%)	1139 (25.6%)	< 0.001
Asthma	448 (5.5%)	282 (6.3%)	0.052
Depression	566 (6.9%)	483 (10.9%)	< 0.001
Peripheral vascular disease	590 (7.2%)	398 (9.0%)	< 0.001
Previous revascularization	749 (9.2%)	604 (13.6%)	< 0.001
Congestive heart failure	331 (4.1%)	283 (6.4%)	< 0.001
Hyperthyroidism	102 (1.3%)	15 (0.3%)	< 0.001
Aortic stenosis	119 (1.5%)	86 (1.9%)	0.045
**Laboratory tests**			
Hemoglobin	138.70 ± 18.67	135.66 ± 20.66	< 0.001
White blood count	10.23 ± 4.83	10.43 ± 4.27	0.025
Sodium	139.03 ± 3.75	138.62 ± 3.93	< .001
Potassium	4.09 ± 0.55	4.11 ± 0.58	0.064
Glucose	9.37 ± 5.21	9.01 ± 4.53	< 0.001
Urea	7.38 ± 4.53	8.13 ± 5.40	< 0.001
Creatinine	103.60 ± 58.77	111.64 ± 72.95	< 0.001

Continuous variables are reported as mean ± standard deviation, while binary variables are reported as N (%).

***Suppressed due to small sample size.

**Table 2:** Baseline characteristics of patients in the CHF derivation and validation samples.

Variable	Derivation sample (N = 7156)	Validation sample (N = 6818)	P-value
**Outcome**			
Discharge systolic blood pressure	124.87 ± 22.27	125.77 ± 21.94	0.017
**Demographic characteristics**			
Age	75.20 ± 11.54	76.23 ± 11.58	< 0.001
Female	3578 (50.0%)	3460 (50.7%)	0.377
**Vital signs on hospital presentation**			
Systolic blood pressure	150.41 ± 33.22	148.42 ± 32.27	< 0.001
Heart rate	94.46 ± 25.30	92.36 ± 25.73	< 0.001
Respiratory rate	25.96 ± 7.25	24.45 ± 6.91	< 0.001
**Signs and symptoms**			
Neck vein distension	3946 (55.1%)	4148 (60.8%)	< 0.001
S3	707 (9.9%)	430 (6.3%)	< 0.001
S4	275 (3.8%)	189 (2.8%)	< 0.001
Rales > 50% of lung field	739 (10.3%)	845 (12.4%)	< 0.001
Pulmonary edema	3691 (51.6%)	4130 (60.6%)	< 0.001
Cardiomegaly	2552 (35.7%)	3014 (44.2%)	< 0.001
**Classic cardiac risk factors**			
Diabetes	2498 (34.9%)	2582 (37.9%)	< 0.001
**Comorbid conditions**			
Cerebrovascular disease/transient ischemic attack	1144 (16.0%)	1223 (17.9%)	0.002
Previous AMI	2637 (36.9%)	2508 (36.8%)	0.936
Atrial fibrillation	2070 (28.9%)	2401 (35.2%)	< 0.001
Peripheral vascular disease	897 (12.5%)	917 (13.4%)	0.108
Chronic obstructive pulmonary disease	1171 (16.4%)	1521 (22.3%)	< 0.001
Dementia	472 (6.6%)	626 (9.2%)	< 0.001
Cirrhosis	51 (0.7%)	52 (0.8%)	0.73
Cancer	802 (11.2%)	759 (11.1%)	0.888
**Findings on electrocardiogram**			
Left bundle branch block	1056 (14.8%)	915 (13.4%)	0.023
**Laboratory tests**			
Hemoglobin	124.17 ± 20.65	123.23 ± 20.53	0.007
WBC (white blood cell) count	9.89 ± 5.23	9.65 ± 4.24	0.003
Sodium	138.37 ± 4.74	138.43 ± 4.86	0.451
Potassium	4.28 ± 0.66	4.26 ± 0.66	0.123
Glucose	9.03 ± 4.69	8.61 ± 4.08	< 0.001
Urea level	10.00 ± 6.32	9.92 ± 6.04	0.458
Creatinine	129.63 ± 94.43	126.42 ± 81.08	0.031

Continuous variables are reported as mean ± standard deviation, while binary variables are reported as N (%).

The use of the data in this project is authorized under Section 45 of Ontario’s Personal Health Information Protection Act (PHIPA) and does not require review by a Research Ethics Board. All research was performed in accordance with relevant guidelines and regulations.

### Methods for predicting discharge systolic blood pressure

We considered six different methods for predicting systolic blood pressure at time of hospital discharge: conventional linear regression estimated using OLS, random forests of regression trees, boosted trees, artificial neural networks, ridge regression, and the lasso. Readers are referred elsewhere for details on these methods^9–14^. The empirical analyses described in this section are motivated by similar analyses conducted in a previous study^7^ with a focus on predicting the probability of the occurrence of a binary outcome. All methods considered all the variables listed in Table 1 as candidate predictor variables. When using OLS regression to predict discharge blood pressure, the regression model included as main effects all the variables. The relationship between discharge blood pressure and each continuous variable was modeled used restricted cubic splines^15^. These six learning methods were selected for two different reasons. First, five of the six (with the exception of neural networks) were included in a recent study comparing the relative performance of different learning methods for predicting binary outcomes^7^. Second, many of these methods have been used in the cardiology literature for predicting patient outcomes^4,16^. Our study may hence be considered a neutral simulation study, where we compare different approaches rather than proposing a new method^17^.

For each disease condition, hyper-parameter tuning was performed in the derivation sample. For both ridge regression and the lasso, the tuning parameter λ was estimated using the cv.glmnet function from the *glmnet* package. This function uses tenfold cross-validation in the derivation sample to select the optimal value of λ. The hyper-parameters were tuned for boosted trees, random forests, neural networks, and OLS regression using a user-derived grid search^18^. The grid had one dimension for OLS regression (number of knots for the restricted cubic splines) and two dimensions for neural networks (number of neurons in the single hidden layer and the weight decay parameter), boosted trees (interaction depth and shrinkage or learning rate) and random forests (number of sampled candidate variables and minimum size of terminal nodes). For a given point on this grid (e.g., for a given number of sampled candidate variables and minimum size of terminal nodes for random forests), the derivation sample was randomly divided into ten approximately equally-sized groups. The given model, with the parameters set to those of the grid point, was fit in nine of the groups. The fitted model was then applied to the remaining group and the predicted discharge blood pressure was obtained for each subject in this remaining group. The accuracy of predictions was quantified using R^2^. This cross-validation process was conducted ten times, so that each of the ten groups was used once for validating predictions. The R^2^ was then averaged across all ten iterations of this procedure. The grid point that resulted in the highest value of the R^2^ was selected for all subsequent applications of that method. For the neural network we allowed a single hidden layer as it has been suggested that this is sufficient for many practical applications^19^ (page 158).

In the AMI sample, the grid searches resulted in the following values for the hyper-parameters: boosted trees (interaction depth: 4; shrinkage/learning rate: 0.065), random forests (number of randomly sampled variables: 6; minimum terminal node size: 20), OLS regression (number of knots: 3), neural networks (5 neurons in the hidden layer, from a grid search that considered the number of neurons ranging from 2 to 15 in increments of 1; weight decay parameter: 0.05), lasso (λ = 0.08596), ridge regression (λ = 0.56553).

In the CHF sample, the grid searches resulted in the following values for the hyper-parameters: boosted trees (interaction depth: 4; shrinkage/learning rate: 0.065), random forests (number of randomly sampled variables: 8; minimum terminal node size: 20), OLS regression (number of knots: 5), neural networks (6 neurons in the hidden layer, from a grid search that considered the number of neurons ranging from 2 to 15 in increments of 1; weight decay parameter: 0), lasso (λ = 0.03323), ridge regression (λ = 0.96881).

Using the hyper-parameters obtained above, each model was fit to the derivation sample (patients hospitalized between 1999 and 2001) and then predictions were obtained for each subject in the validation sample (patients hospitalized between 2004 and 2005). Accuracy of predictions was assessed using three metrics: R^2^, mean squared error (MSE), and mean absolute error (MAE)^20^. R^2^ was computed as the square of the Pearson correlation coefficient between observed and predicted discharge blood pressure, while MSE and MAE were estimated as $\frac{1}{N}\sum\limits_{i = 1}^{N} {(Y_{i} - \hat{Y}_{i} )^{2} }$ and $\frac{1}{N}\sum\limits_{i = 1}^{N} {|Y_{i} - \hat{Y}_{i} |}$, respectively, where $Y$ denotes the observed blood pressure and $\hat{Y}$ denotes the estimated blood pressure.

For all methods, we used implementations available in R statistical software (R version 3.6.1, R Foundation for Statistical Computing, Vienna, Austria). For random forests we used the randomForest function from the *randomForest* package (version 4.6-14). The number of trees (500) was the default in this implementation. For boosted trees we used the gbm function from the *gbm* package (version 2.5.1). The number of trees (100) was the default in this implementation. We used the ols and rcs functions from the *rms* package (version 5.1-3.1) to estimate the OLS regression model incorporating restricted cubic regression splines. Feed-forward (or multilayer perceptron) neural networks with a single hidden layer were fit using the *nnet* package (version 7.3-12) with a linear activation function. Ridge regression and the lasso were implemented using the functions cv.glmnet (for estimating the λ parameter using tenfold cross-validation) and glmnet from the *glmnet* package (version 2.0-18).

### Results of empirical analyses

**Figure 1:**
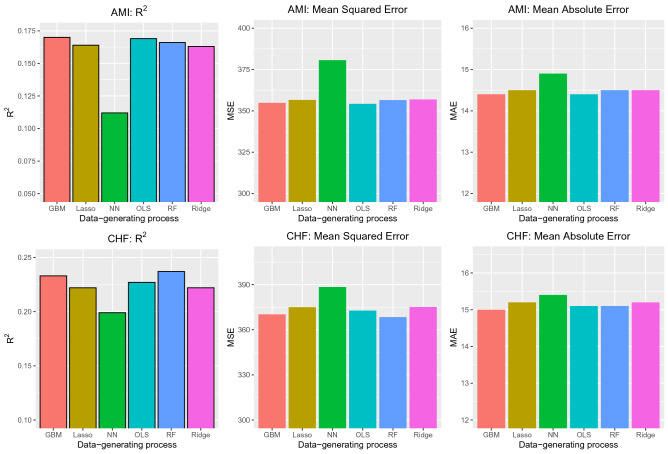
Performance in validation sample (Case study).

The performance of the six different methods for predicting discharge blood pressure in the validation sample (patients hospitalized between 2004 and 2005) are summarized in Fig. 1. In the AMI sample, boosted trees resulted in predictions with the highest R^2^ (0.17); however, differences between five of the six methods were minimal (range: 0.163 to 0.17 for five of the six methods). Note that R^2^ is reported as a proportion: the proportion of the variation in discharge blood pressure that is explained by the model. OLS regression resulted in estimates with the lowest MSE, while both OLS regression and boosted trees resulted in estimates with the lowest MAE. As with R^2^, MSE and MAE did not vary meaningfully across five of the six methods. The performance of the neural network differed from that of the other five across all three performance metrics.

In the CHF sample, random forests resulted in predictions with the highest R^2^ (23.7%); however, differences between five of the six methods were minimal again (range: 22.2 to 23.7%). Random forests resulted in estimates with the lowest MSE, while boosted trees resulted in estimates with the lowest MAE. As with R^2^, MAE did not vary meaningfully across five of the six methods (range: 15.0 to 15.2). As in the AMI sample, the neural network had substantially worse performance than the other five methods across all three metrics.

When comparing the three linear model-based approaches, neither of the two penalized approaches (lasso and ridge regression) had an advantage over conventional OLS regression in either disease samples. In both diseases, the lasso and ridge regression had very similar performance to each other.

In conclusion, in these empirical analyses, a tree-based machine learning method (either boosted trees or random forest) tended to result in estimates with the greatest predictive accuracy in the validation samples. However, differences between five of the methods were minimal. Neural networks resulted in estimates with substantially worse performance compared to the other five methods.

## Monte Carlo simulations for comparing the relative performance of different prediction methods

In the preceding section we observed that the best-performing method varied between the two diseases and that there were minimal differences between the five of the six prediction methods. In the current section, we examine the influence of how outcomes are generated on the relative performance of the different prediction methods. We considered six different data-generating processes, each based on one of the six models fit in the previous section. Using the samples described above, we generate outcomes in each of the derivation and validation samples using the given data-generating process. We then fit each of the six modeling methods to the derivation samples and then apply the fitted model to the simulated validated sample to assess the performance of each method. This process of simulating data using a given learning method is similar to one that was recently used to compare the accuracy of different methods for predicting binary outcomes^7^.

### Six data-generating processes for simulating outcomes

We considered six different data-generating processes for each of the two diseases (AMI and CHF). We describe the approach in detail for the AMI sample. An identical approach was used with the CHF sample. We used the derivation and validation samples described in the empirical analyses above. We made one modification to the validation samples described above. The validation sample used above consisted of 4444 subjects (AMI validation sample) and 6818 (CHF validation sample). In order to remove variation in external performance due to small sample sizes, we sampled with replacement from each validation sample to create validation samples consisting of 100,000 subjects. For a given learning method (e.g., random forests), the method was fit in the derivation sample. The fitted model was then applied to both the derivation sample and the validation sample. Using the model/algorithm fit in the derivation sample, a predicted outcome (discharge systolic blood pressure) was obtained for each subject in each of the two datasets (derivation and validation samples). For random forests, boosted trees, neural networks, the lasso and ridge regression, we proceeded as follows: Using these predicted blood pressures at discharge, a continuous outcome was simulated for each subject as follows. First, for each subject in the derivation sample, a residual or prediction error was computed as the difference between the true observed discharge blood pressure and the estimated blood pressure obtained from the fitted model. Second, for each subject in the derivation sample, a residual was drawn with replacement from the empirical distribution of residuals estimated in the previous step. Third, the sampled residual was added to the estimated discharge blood pressure. This quantity is the simulated outcome for the given patient. This process was then repeated in the validation sample to obtain a simulated outcome for each subject in the validation sample. Note that the given prediction model was only fit once (in the derivation sample) but was then applied in both the derivation and validation samples to obtain estimated values of discharge blood pressure. These simulated outcomes were then used as the ‘true’ outcomes in all subsequent analyses. The above process was used when the data-generating process was based on random forests, boosted trees, neural networks, the lasso, and ridge regression. When the data-generating process was based on OLS regression, we used a modified version of this process. Instead of sampling from the empirical distribution of residuals, we sampled residuals from a normal distribution with mean zero and standard deviation equal to that estimated for error distribution from the OLS model. These sampled residuals were then added to the estimated discharge blood pressure to produce simulated continuous outcomes.

Using the process described above, the simulated outcomes reflected the multivariable relationship between the baseline covariates and the outcome that was implied by the fitted algorithm (e.g., random forests). This process was repeated 1000 times, resulting in 1000 pairs of derivation and validation samples. This process was repeated for each of the six different statistical/machine learning methods. Thus, we had a data-generating process based on boosted trees, random forests, neural networks, the lasso, ridge regression, and OLS regression. This approach to simulating outcomes is similar to that employed in our recent paper examining the relative accuracy of different methods for estimating probabilities^7^.

### Performance of different predictive methods under different data-generating processes

For a given pair of derivation and validation samples, we fit each of the six statistical/machine learning methods (boosted trees, random forests, neural networks, the lasso, ridge regression, and OLS regression) in the derivation sample and then applied the fitted model to the validation sample. In the validation sample, we obtained, for each subject, an estimated discharge blood pressure for each of the six prediction methods. The performance of the predictions obtained using each method was assessed using the three metrics described above (R^2^, MSE, and MAE). Thus, for a given data-generating process and a given prediction method we obtained 1000 values of R^2^, MSE and MAE.

Thus, when outcomes were simulated in the derivation and validation samples using random forests, we assessed the predictive accuracy of boosted trees, random forests, neural networks, the lasso and ridge regression, and OLS regression. This process was repeated using the datasets in which outcomes were simulated using the five other data-generating processes.

### Results of the simulations

#### AMI sample

**Figure 2:**
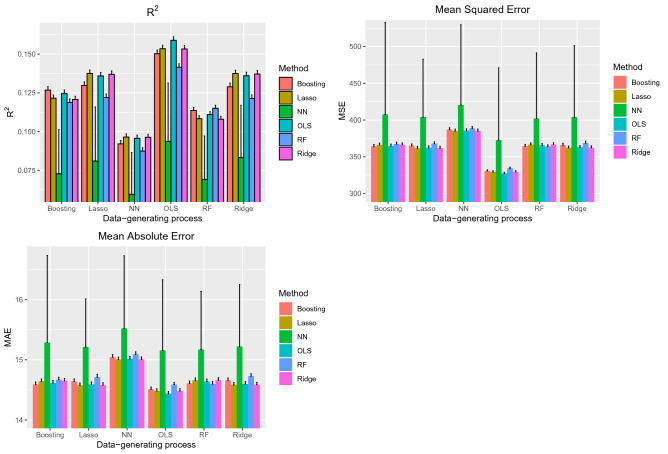
Performance in AMI sample (External validation).

The performance of the six prediction methods under the six different data-generating processes is reported in Fig. 2. There is one panel for each of the three performance metrics. For each performance metric, we summarize the results across the 1000 simulation replicates using bar charts, with one bar for each combination of data-generating process and analytic method. An error bar denoting the standard deviation of the performance metric across the 1000 simulation replicates has been added to each bar.

Across all six data-generating processes and across all three performance metrics, the use of neural networks tended to result in predictions with the lowest accuracy. Even when outcomes were simulated using a neural network, the other five methods tended to result in predictions with higher accuracy than did the use of neural networks. The difference in performance between neural networks and that of the other five methods was substantially greater than the differences amongst the other five methods.

When outcomes were generated using boosted trees, the use of boosted trees tended to result in estimates with the highest R^2^, while estimates obtained using OLS regression tended to result in estimates with comparable performance. When outcomes were generated using an OLS regression model, the use of OLS regression tended to result in estimates with the highest R^2^. The performance of OLS regression was followed by that of boosted trees and the two penalized regression methods. When outcomes were generated using a penalized regression method, the three linear regression models tended to result in estimates with the highest R^2^. Finally, when outcomes were generated using random forests, the use of boosted trees and random forests tended to result in estimates with the highest R^2^. When considering the three linear regression-based approaches, there was no advantage to using a penalized regression approach compared to using OLS regression. When assessing accuracy using MSE or MAE, the differences between the five non-neural network approaches tended to be minimal. In particular, regardless of the data-generating processes, the use of OLS regression tended to perform well, and there were no meaningful benefits to using a different approach. MSE and MAE of estimates obtained using neural networks displayed high variability across the 1000 simulation replicates.

#### CHF sample

**Figure 3:**
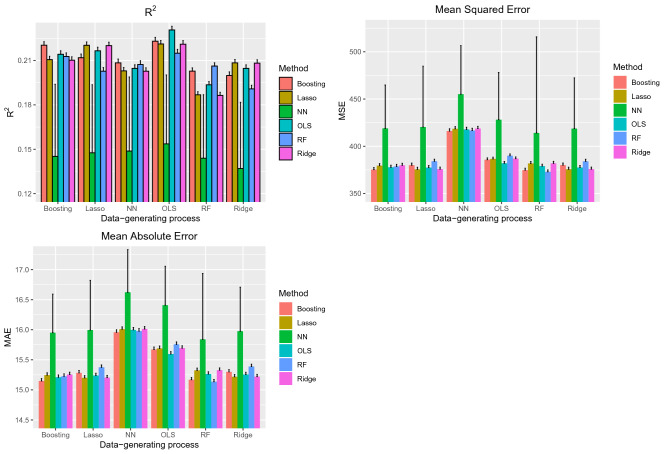
Performance in CHF sample (External validation).

The performance of the six different prediction methods under the six data-generating processes are reported in Fig. 3. As in the AMI sample, the most obvious observation is the poor performance of neural networks compared to that of the other five methods. This was true across all six data-generating process and all three performance metrics. Similarly, as in the AMI sample, the difference in performance between neural networks and that of the other five methods was substantially greater than the differences amongst the other five methods.

When outcomes were generated using boosted trees, the use of boosted trees tended to result in estimates with the highest R^2^. Similarly, when outcomes were simulated using random forests, the use of random forests tended to result in estimates with the highest R^2^, although the performance of boosted trees was comparable. When outcomes were generated using a linear regression-based approach, then the three linear regression-based approaches tended to result in estimates with the highest R^2^. Similar results were observed when MSE and MAE were used to assess performance accuracy. As in the AMI sample, when considering the three linear regression-based estimation methods, there were rarely meaningful benefits to using a penalized estimation method compared to using OLS regression.

## Discussion

There is a growing interest in comparing the relative performance of different machine and statistical learning methods for predicting patient outcomes. To better understand differences in the relative performance of competing learning methods for predicting continuous outcomes, we used two empirical comparisons and Monte Carlo simulations using six different data-generating processes, each based upon a different learning method. These simulations enabled us to examine the performance of methods different from those under which the data were generated compared to the method that was used to generate the data. In both of the empirical analyses and in all six sets of Monte Carlo simulations, the performance of neural networks was substantially poorer than that of the other five learning methods.

There is a limited number of studies assessing the performance of machine learning methods for modeling blood pressure. Using a sample of 400 subjects, Golino et al.^21^ used classification trees to identify subjects with elevated blood pressure (systolic blood pressure > 120.0 mmHg for women and systolic blood pressure > 140.0 mmHg for men; note that in this application blood pressure was dichotomized). Sex-stratified analyses were conducted with split-sample validation. They found that the predictive accuracy of classification trees was slightly greater than that of logistic regression. Alkaabi et al.^22^ compared the performance of classification trees, random forests, and logistic regression for identifying subjects with hypertension (systolic blood pressure ≥ 140 mmHg and/or a diastolic pressure ≥ 90 mmHg or use of hypertension medication). Using 987 subjects and fivefold cross-validation, they found that the three methods tended to have similar performance across an array of metrics. Using a sample of 498 subjects and a split-sample validation approach, Wu et al.^23^ used artificial neural networks to predict blood pressure (as a continuous variable) and found that approximately half of subjects had an estimated blood pressure that was within 10 mmHg of the observed blood pressure. Using data on 18 subjects, each of whom had their blood pressure assessed on multiple occasions, Zhang et al.^24^ used both split-sample and tenfold cross-validation to compare the performance of support vector machines, neural networks, and linear regression to predict subjects’ blood pressure, and found that support vector machines had the greatest predictive accuracy. These earlier studies differ from our empirical analyses in two important ways. First, these studies used split-sample validation or K-fold cross-validation, both of which are forms of internal validation. In comparison, we used an independent validation sample from a different temporal period. Thus we examined the historical transportability of our predictions, which is a stronger form of validation than internal validation^25^. Second, the number of subjects in both of our derivation samples and in both of our validation samples were substantially higher than those used in these previous studies.

An advantage to the current study was its use of simulations to compare the relative performance of different learning methods for predicting blood pressure. A strength of the design of these simulations is that they were based on two real data sets, each with a realistic correlation structure between predictors and with realistic associations between predictors and outcomes. Thus, we were able to simulate datasets reflective of those that would be seen in specific clinical contexts. Importantly, both the sizes of the simulated dataset and the number of predictors that we considered are reflective of what is often encountered in clinical research. Some might argue that the number of predictors (33 and 28 in the AMI and CHF studies respectively) is relatively high for conventional regression modeling, and relatively low for modern machine learning techniques.

There is a paucity of studies that have used simulations to compare the performance of statistical learning methods with that of ML methods for prediction from a more or less neutral position^2^. In a recent study, we used simulations similar in design to those described in the current study to compare the performance of different learning methods to predict binary outcomes^7^. In that earlier study, we found that logistic regression and boosted trees tended to have superior performance to the other methods across a range of data-generating processes and performance metrics. In a study using simulations similar to ours, Van der Ploeg et al.^26^ compared the number of events per variable that were required to achieve estimates of c-statistics with minimal optimism for different statistical and ML methods. In a simulation-based study, Kirasich et al.^27^ found that logistic regression resulted in classifications with higher accuracy than did random forests. Finally, in a simulation-based study that compared a set of classification methods to the boosted version of each classifier, Vafeiadas et al.^28^ found that, for each classifier, the use of boosting resulted in improved performance.

The objective of the current study was not to develop a new learning method nor was it to improve existing learning methods^17^. Our objective was to compare the relative performance of different learning methods for predicting a continuous outcome. As noted above, while there is a growing number of studies comparing different learning methods, the large majority of these studies rely on empirical comparisons using a single dataset. A strength of the current study is its use of Monte Carlo simulations to conduct these comparisons systematically. A methodological contribution of the current study is providing a framework for Monte Carlo simulations that allows for a more informed comparison of different learning methods. Because we knew which learning method was the true model that generated the outcomes, the performance of each of the other five methods could be compared to that of the true method. For example, we demonstrated that when outcomes were generated using boosted trees, the use of OLS regression had performance comparable to that of boosted trees for predicting blood pressure (in the AMI sample).

An advantage to regression-based approaches to predicting blood pressure is that the estimated coefficients have a simple interpretation: the mean change in blood pressure associated with a one-unit change in the given covariate. Furthermore, by publishing the regression coefficients, the investigators can allow anyone to compute the expected blood pressure for a given covariate pattern. In contrast, machine learning methods have often been criticized as ‘black boxes’, and it is difficult to assess the effect of specific covariates on the outcome. Furthermore, it is difficult to publish the resultant model in such a way as to allow others to compute expected blood pressure for specific covariate patterns in independent validation studies^29^.

In conclusion, we found that a default implementation of a neural network had substantially poorer performance compared to five other learning methods for predicting discharge systolic blood pressure in patients hospitalized with heart disease. This finding was observed both in two sets of empirical analyses and in six sets of Monte Carlo simulations. We also observed that there was no meaningful advantage to the use of penalized linear models (i.e., the lasso or ridge regression) compared to using OLS regression. Boosted trees tended to have the best performance of the different machine learning methods for the number of covariates studied. Investigators interested in predicting blood pressure may often be able to limit their attention to OLS regression and boosted trees and select the method that performs best in their specific context. We encourage researchers to apply our simulation framework to other diseases and other empirical datasets to examine whether our findings persist across different settings and diseases.

### Ethics declarations

The use of data in this project was authorized under Section 45 of Ontario’s *Personal Health Information Protection Act*, which does not require review by a Research Ethics Board. This study did not include experiments involving human subjects or tissue samples.

## Data Availability

The data sets used for this study were held securely in a linked, de-identified form and analysed at ICES. While data sharing agreements prohibit ICES from making the data set publicly available, access may be granted to those who meet pre-specified criteria for confidential access, available at www.ices.on.ca/DAS. If you are interested in requesting ICES Data & Analytic Services, please contact ICES DAS (e-mail: das@ices.on.ca or at 1-888-480-1327).
